# Unravelling longitudinal associations of social and emotional loneliness with social isolation and mental health outcomes: a cross-lagged panel network analysis

**DOI:** 10.1017/S2045796025100383

**Published:** 2026-01-02

**Authors:** Błażej Misiak, Ernest Tyburski, Agnieszka Samochowiec, Jerzy Samochowiec

**Affiliations:** 1 Department of Psychiatry, Wroclaw Medical University, Wroclaw, Poland; 2 Department of Psychiatry, Pomeranian Medical University, Szczecin, Poland; 3 Department of Clinical Psychology and Psychoprophylaxis, Institute of Psychology, University of Szczecin, Szczecin, Poland

**Keywords:** community mental health, social disconnection, social factors, social functioning, social network

## Abstract

**Aims:**

Loneliness is a global public health concern that has been widely associated with a variety of mental health impairments. Two dimensions of loneliness have been differentiated, that is, social loneliness (the perceived absence or inadequacy of a broader social network) and emotional loneliness (the perceived absence of a close, intimate relationship or emotional support from a significant person). The present study aimed to test the hypothesis that both dimensions of loneliness are differentially associated with mental health outcomes.

**Methods:**

Altogether, 3275 individuals (aged 45.2 ± 15.7 years, 47.9% men), enrolled from the general population, were assessed at two waves spanning 6 to 7 months. Social and emotional loneliness were quantified using the 11-item De Jong Gierveld Loneliness Scale. Social isolation was assessed with the six-item Lubben Social Network Scale, depressive symptoms with the Patient Health Questionnaire-9, generalised anxiety with the Generalised Anxiety Disorder-7, social anxiety with the Social Interaction Anxiety Scale, and paranoid ideation with the Revised Green et al. Paranoid Thoughts Scale. The data were analysed using a cross-lagged panel network model. Covariates included age, gender, education, employment status, place of residence, monthly income, the history of psychiatric treatment and substance use.

**Results:**

Both dimensions of loneliness were bidirectionally associated and were found to have the highest overall weight of outcoming network connections. Emotional loneliness was bidirectionally and positively associated with all measures of mental health. In turn, social loneliness predicted higher levels of social anxiety but was not associated with other mental health outcomes. It was bidirectionally associated with social isolation.

**Conclusions:**

The findings imply the relevance of differentiating social and emotional dimensions of loneliness in the assessment of its underlying mechanisms and consequences for mental health. Emotional loneliness might show a greater importance in the development of psychopathological symptoms compared to its social dimension.

## Introduction

Loneliness is increasingly being recognised as a global public health concern due to its high prevalence and negative impact on all aspects of health, development and wellbeing (Iks, 2024; Park *et al.*, 2020). It might be defined as a distressing experience rooted in the perceived discrepancy between desired and actual social connections (Hawkley and Cacioppo, 2010). It is not the same as social isolation, representing an objective lack of social connections or interactions (Cacioppo and Hawkley, 2009; Holt-Lunstad *et al.*, 2015). In this regard, both constructs, although interconnected, may emerge as independent experiences. For instance, some individuals might have several social connections while experiencing loneliness.

It has been noted that loneliness is not a homogeneous construct. It might manifest either as social or emotional loneliness (Weiss, 1973). The first one is the perceived absence or inadequacy of a broader social network. In turn, the second one is the perceived absence of a close, intimate relationship or emotional support from a significant person. According to Weiss (1973), who was the first to formally differentiate both facets of loneliness, social and emotional loneliness originate from the absence of fundamentally different types of relationships, that is, attachment vs integration. Previous studies have shown that social and emotional loneliness demonstrate weak-to-moderate correlations supporting the notion that they represent distinct constructs (Dahlberg and McKee, 2014; Green *et al.*, 2001; Russell *et al.*, 1984).

Loneliness has been robustly associated with depression, anxiety disorders and psychosis, together with their subclinical manifestations, investigated in general population samples. A recent meta-analysis estimated the prevalence of loneliness among individuals with any diagnosis of severe mental disorders (either depression, bipolar disorder, schizoaffective disorder or schizophrenia) at 59.1% (Hajek *et al.*, 2025). In this meta-analysis, depressive symptoms and subjective well-being emerged as the major factors contributing to loneliness across included studies. Depression and its symptoms represent mental health outcomes most widely investigated with respect to loneliness. Previous studies have found that their associations with loneliness are bidirectional and occur across all age groups (Chen *et al.*, 2023). Similarly, the association of social (Maes *et al.*, 2019) and generalised (Wilkialis *et al.*, 2021) anxiety with loneliness is well-documented. Finally, several studies have found the association of loneliness with the psychosis continuum as reported by a recent meta-analysis by Chau *et al.* (2019). Importantly, this meta-analysis demonstrated that this association shows higher effect size estimates for paranoid ideation and also appears to be stronger in non-clinical samples.

Less is known about the differential associations of social and emotional loneliness with mental health characteristics. A recent cross-sectional analysis of data from university students demonstrated that social loneliness is most strongly explained by social isolation, while emotional loneliness might be most strongly explained by depressive and social anxiety symptoms (Wolters *et al.*, 2023). Generalised anxiety was found to be linked to loneliness through the effects of depressive symptoms. Another network analysis was also carried out in university students and was based on longitudinal data (van den Bulck *et al.*, 2025). The authors found that chronic emotional loneliness is associated with depressive and social anxiety symptoms, while chronic social loneliness is related to lower levels of secure attachment style. However, another study of high-school students showed that both social and emotional loneliness are associated with depressive and anxiety symptoms (Dziedzic *et al.*, 2021). Finally, one study performed in a clinical sample revealed higher levels of emotional, but not social, loneliness among individuals with late-life depression (Ngan and Cheng, 2024). Moreover, in this study, emotional loneliness was found to predict suicidal ideation and mediated the association of somatic comorbidity with depression.

Taken together, previous studies have suggested that social and emotional loneliness might be differentially associated with mental health outcomes, with the latter dimension being more closely associated. However, existing evidence is scarce and originates from studies based on student populations and late-life depression. Therefore, the potential to generalise findings to other populations might be limited. It is also important to note that these studies do not inform about the temporal ordering of loneliness dimensions and mental health outcomes. Finally, the analysis of mental health outcomes was limited to depressive and anxiety symptoms, while neglecting other dimensions of psychopathology.

To bridge existing research gaps, the present study aimed to investigate longitudinal associations of social and emotional loneliness with social isolation and a range of mental health outcomes, including depressive symptoms, generalised anxiety, social anxiety and paranoid ideation. Importantly, the study was conducted in a non-clinical, community-based sample in order to capture the full continuum of loneliness and symptom severity. This design makes it possible to detect subclinical variations that are often overlooked in clinical settings but may represent important precursors of later disorder onset. For this reason, all mental health outcomes were analysed as continuous scores, allowing for a more fine-grained assessment of associations across the spectrum rather than restricting analyses to diagnostic thresholds. We hypothesised that loneliness shows bidirectional associations with mental health outcomes and that these associations are stronger in the case of emotional loneliness.

## Method

### Participants

Participants were enrolled using a quota-based approach to ensure representativeness across key sociodemographic variables such as age, gender, education level, employment status and geographic location. Data collection was conducted via self-administered online surveys. Baseline measurements were obtained between July and August 2024, and a follow-up survey was administered in February 2025. At both stages, participants were recruited through a research panel managed by a professional company, which maintains an online pool of verified individuals in Poland. This panel, consisting of over 70 000 individuals from all regions of the country, is regularly updated through outreach initiatives.

Eligibility was limited to adults aged 18 years and older. Participants who completed both waves of the survey received compensation equivalent to 10 EUR. To maintain data quality, several reliability checks were employed. Respondents were excluded if they: (1) completed the survey too quickly (under 30% of the median duration); (2) failed attention checks (e.g., missed instructions to select a specific option); (3) provided inconsistent answers to repeated items; or (4) entered nonsensical responses such as random character strings. The study protocol was approved by the Bioethics Committee at Wroclaw Medical University (approval number: 553/2024), and all participants provided digital informed consent prior to participation.

### Assessments

Participants were assessed by means of self-reports covering the following constructs: (1) loneliness; (2) social isolation; (3) depressive symptoms; (4) generalised anxiety; (5) social anxiety and (6) paranoid ideation.

### Loneliness

To assess loneliness, the 11-item version of the De Jong Gierveld Loneliness Scale (DJGLS) was used (de Jong-gierveld and Kamphuls, 1985; Grygiel *et al.*, 2013). Respondents answered each item using a five-point scale: ‘yes!’, ‘yes’, ‘more or less’, ‘no’ and ‘no!’. The scale measures loneliness across two dimensions: emotional loneliness (six items) and social loneliness (five items). The scoring method involves counting affirmative and neutral responses (‘yes!’, ‘yes’, ‘more or less’) on emotional loneliness items, and negative and neutral responses (‘no!’, ‘no’, ‘more or less’) on social loneliness items. The final score ranges from 0 to 11, with higher values indicating increased loneliness. In the current sample, the emotional and social subscales had baseline Cronbach’s alpha values of 0.874 and 0.810, respectively.

### Social isolation

Social isolation was measured using the 6-item version of the Lubben Social Network Scale (LSNS-6) (Lubben *et al.*, 2006). The scale assesses how many friends and family members participants interact with at least monthly, can confide in, or seek support from. Responses are given on a six-point Likert scale. The total score varies from 0 to 30, where higher scores indicate a greater social network density. To reflect the level of social isolation, the total score of LSNS-6 was reverse-coded. In the current study, the Cronbach’s alpha was 0.876 for baseline data.

### Depressive symptoms

The assessment of depressive symptoms was conducted using the Patient Health Questionnaire-9 (PHQ-9) (Kroenke *et al.*, 2001). The tool consists of nine items referring to depressive symptoms over the prior 2 weeks. Each item is rated on a four-point scale ranging from zero (‘not at all’) to three (‘nearly every day’). Total scores span from 0 to 27, with higher scores denoting more severe depressive symptomatology. In the current study, the Cronbach’s alpha was 0.897 for baseline data.

### Generalised anxiety

Symptoms of generalised anxiety were measured using the Generalised Anxiety Disorder-7 (GAD-7) scale (Spitzer *et al.*, 2006). This seven-item questionnaire evaluates anxiety symptoms experienced over the preceding 2 weeks, with a response range from zero (‘not at all’) to three (‘nearly every day’). The possible total score ranges from 0 to 21, where higher values represent more pronounced symptoms. In the current study, the Cronbach’s alpha was 0.944 for baseline data.

### Social anxiety

To evaluate social anxiety, the Social Interaction Anxiety Scale (SIAS) was applied (Mattick and Clarke, 1998). It includes 20 items reflecting various social anxiety characteristics. Participants rate the extent to which each statement applies to them on a scale from zero (‘not at all’) to four (‘extremely’). In the current sample, the Cronbach’s alpha was 0.947 at baseline.

### Paranoid ideation

Paranoid ideation was assessed using the Revised Green et al. Paranoid Thoughts Scale (R-GPTS) (Freeman *et al.*, 2021). This scale includes two sections: Part A (eight items) measures ideas of reference, and Part B (10 items) measures ideas of persecution, all within the past month. Responses range from zero (‘not at all’) to four (‘totally’). Participants are asked to rate experiences excluding those influenced by substance use. The Cronbach’s alpha for the two subscales in the current study was 0.930 (Part A) and 0.964 (Part B) at baseline.

### Data analysis

Participant data that did not pass quality control or involved incomplete survey submissions were excluded from analysis. As a result, the final dataset did not contain any missing values for the included items. Initially, comparisons were made between participants who completed both waves of data collection (referred to as completers) and those who did not complete the follow-up (non-completers). Independent *t*-tests were used for continuous variables, and chi-squared tests were used for categorical data. Statistical significance was set at *p* < 0.05. All analyses were conducted using SPSS version 28.

Subsequently, cross-lagged panel network (CLPN) modelling was used to analyse both autoregressive and cross-lagged effects. The CLPN approach was applied as it allows simultaneous modelling of multiple variables and their reciprocal influences over time while visualising these associations as a network (Freichel *et al.*, 2023; Wysocki *et al.*, 2023). A key strength of this method is that it integrates the temporal ordering of effects (via cross-lagged paths) with a network framework, enabling identification of central variables that may drive change within the system. CLPN thus might provide a more nuanced picture of the interplay between loneliness and mental health outcomes than traditional pairwise cross-lagged models.

Variables representing emotional loneliness, social loneliness, social isolation, depressive symptoms, generalised anxiety, social anxiety and paranoid ideation at baseline and follow-up were modelled as network nodes. Covariates included age, gender, education level, employment status, place of residence, monthly income, the lifetime history of psychiatric treatment and use of substances other than alcohol or nicotine in the preceding month. Analyses were conducted in R, using the *glmnet* package (Friedman *et al.*, 2010) for regression modelling and the *qgraph* package for network visualisation (Epskamp *et al.*, 2012). Least Absolute Shrinkage and Selection Operator (LASSO) regressions were applied to detect cross-lagged and autoregressive relationships while minimising the influence of weak connections.

Node importance within the symptom network was evaluated using strength centrality metrics (Jones *et al.*, 2018). Strength centrality represents the total weight of edges connecting a node to other nodes in the network. Specifically, out-strength refers to the total weight of edges from a node to other nodes, while in-strength captures the sum of weights from other nodes directed toward the node of interest.

To assess the accuracy and stability of the estimated network, bootstrapping methods (both non-parametric and case-drop) were implemented using the *bootnet* package (Epskamp *et al.*, 2018). Network stability was interpreted using the correlation stability coefficient (CS-C), which reflects the maximum proportion of data that can be omitted while maintaining a correlation above 0.70 between the original and subset networks. The CS-C value greater than 0.25 is considered acceptable (Epskamp and Fried, 2018). Bootstrapping was also applied to evaluate differences in edge weights and centrality indices across the network.

## Results

### General characteristics of the cohort

Altogether, 10 985 individuals were approached for participation (**Supplementary Fig. 1).** Of these, 6702 agreed to participate, yielding a raw response rate of 61.5%. A total of 1603 individuals were subsequently excluded prior to the baseline dataset: 490 initiated but did not complete the survey, 828 were interrupted while completing the predefined quota, and 285 failed accuracy checks (including 121 individuals with implausibly fast completion times). This resulted in 5099 individuals completing the baseline assessment (46.8% of all invited). At follow-up, 3275 individuals participated, corresponding to a retention rate of 64.2%.

To evaluate potential selection bias, individuals who completed both assessments and those who dropped out after baseline were compared. No significant differences were observed in sociodemographic or clinical characteristics (Table 1).

**Table 1.** Descriptive characteristics of the sample

	Total sample (*n* = 5099)	Completers (*n* = 3275)	Non-completers (*n* = 1824)	*P*
Age, years	44.9 ± 15.4	45.2 ± 15.7	44.8 ± 15.2	0.189
Gender, men	2431 (47.7)	1568 (47.9)	863 (47.3)	0.718
Education				
Primary	94 (1.8)	53 (1.6)	41 (2.2)	0.858
Vocational	411 (8.1)	273 (8.3)	138 (7.6)	
Secondary	2098 (41.1)	1345 (41.1)	753 (41.3)	
Higher	2496 (49.0)	1604 (49.0)	892 (48.9)	
Employment				
Unemployed	435 (8.5)	265 (8.1)	170 (9.3)	0.406
Retired/disability pension	1002 (19.6)	632 (19.3)	370 (20.3)	
Student	234 (4.6)	156 (4.8)	78 (4.3)	
Part-time work	482 (9.5)	317 (9.7)	165 (9.0)	
Full-time work	2946 (57.8)	1905 (58.1)	1041 (57.1)	
Place of residence				
Rural	1650 (32.4)	1065 (32.5)	585 (32.1)	0.991
Urban, <50 000 inhabitants	1166 (22.9)	750 (22.9)	416 (22.8)	
Urban, 50 000–150 000 inhabitants	816 (16.0)	525 (16.0)	291 (16.0)	
Urban, 150 000–500 000 inhabitants	714 (14.0)	457 (14.0)	257 (14.0)	
Urban, >500 000 inhabitants	753 (14.7)	478 (14.6)	275 (15.1)	
Monthly income				
<750 USD	1082 (21.1)	695 (21.2)	387 (21.2)	0.170
750–1500 USD	2485 (48.7)	1621 (49.6)	864 (47.4)	
1500–2500 USD	722 (14.2)	468 (14.3)	254 (13.9)	
2500–3750 USD	133 (2.6)	80 (2.4)	53 (2.9)	
>3750 USD	44 (0.9)	31 (0.9)	13 (0.7)	
Refused to answer	633 (12.4)	380 (11.6)	253 (13.9)	
Psychiatric treatment				
Lifetime	1108 (21.7)	705 (21.5)	403 (22.1)	0.638
Preceding month	539 (10.6)	327 (10.0)	512 (28.1)	0.068
Substance use, preceding month^a^	353 (6.9)	213 (6.5)	140 (7.7)	0.353
Depressive symptoms, PHQ-9	7.4 ± 6.0	7.3 ± 5.8	7.6 ± 6.1	0.078
Generalised anxiety, GAD-7	6.1 ± 5.5	5.9 ± 5.3	6.2 ± 5.6	0.068
Social anxiety, SIAS	30.7 ± 16.1	31.0 ± 16.3	30.0 ± 16.0	0.198
Paranoid thoughts, R-GPTS	16.9 ± 17.2	16.5 ± 17.0	17.0 ± 17.5	0.100
Social isolation, LSNS-6^b^	15.9 ± 6.0	16.0 ± 6.5	15.8 ± 5.8	0.228
Social loneliness, DJGLS	2.5 ± 1.8	2.4 ± 1.8	2.6 ± 1.8	0.219
Emotional loneliness, DJGLS	2.7 ± 2.3	2.7 ± 2.3	2.8 ± 2.3	0.258

Data are reported as mean ± SD or *n* (%)

a Except for nicotine and alcohol. ^b^Reversed coding was used to show the level of social isolation.

*Note*: DJGLS, the De Jong Gierveld Loneliness Scale; GAD-7, the Generalized Anxiety Disorder-7; LSNS-6, the Lubben Social Network Scale-6; PHQ-9, the Patients Health Questionnaire-9; R-GPTS, the Revised Green et al. Paranoid Thoughts Scale; SIAS, the Social Interaction Anxiety Scale.

### Network estimation

**Figure 1.**
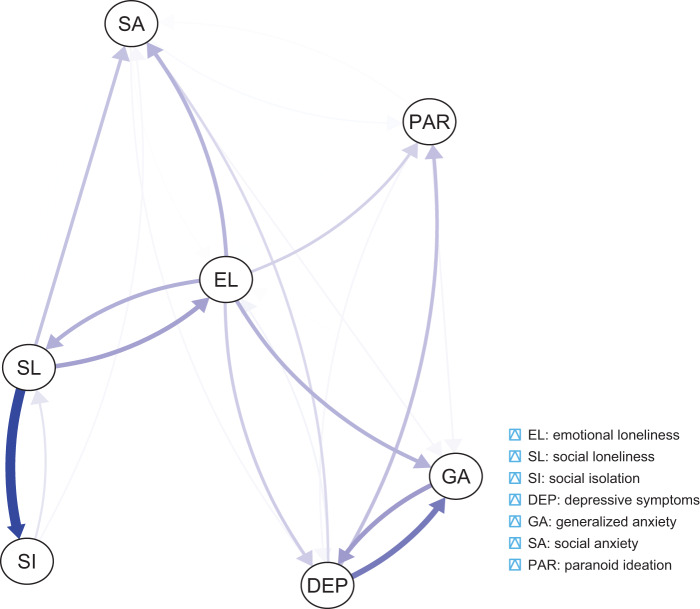
The network estimated in the present study. Thicker and more saturated arrows reflect shorter cross-lagged associations. To ease visual interpretation, auto-regressive effects are not shown.

**Figure 2.**
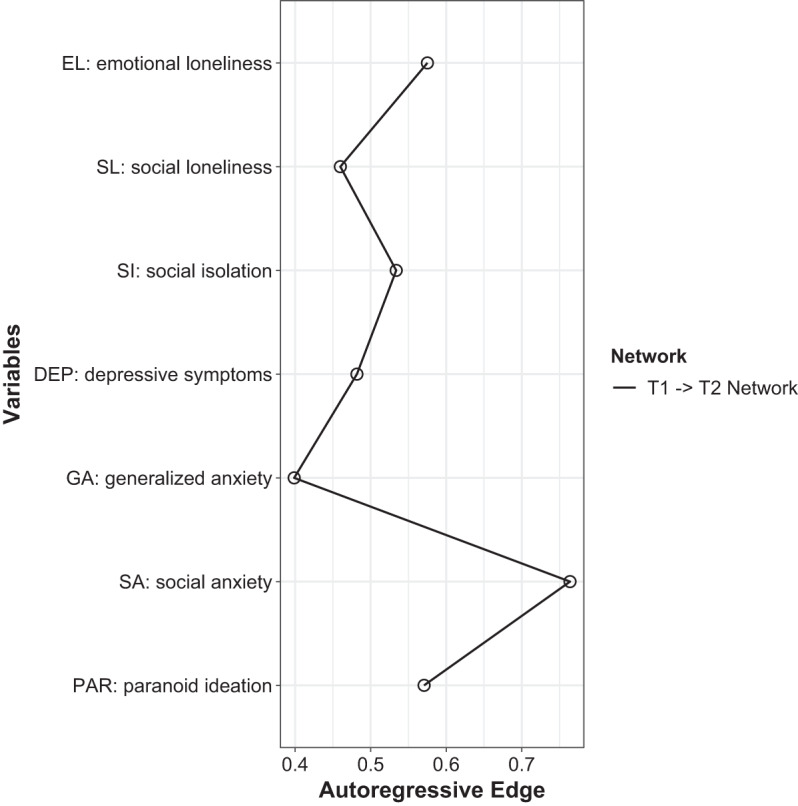
Auto-regressive effects.

The network analysed in the present study is shown in Fig. 1. A total of 25 cross-lagged effects were found to have non-zero weights (out of 42 potential cross-lagged effects, 59.5%, Supplementary Table 1, Supplementary Fig. 2). The strongest connection led from social loneliness to social isolation (weight = 0.311), while the weakest one led from social anxiety to social isolation. The cross-lagged effect for the path from social loneliness to social isolation had a significantly higher weight compared to almost all other cross-lagged effects in the network (except for the one from emotional loneliness to paranoid ideation). Auto-regressive effects are plotted in Fig. 2. The greatest autoregressive effect was found for social anxiety, while the smallest one was observed for generalised anxiety.

Social and emotional loneliness were bidirectionally associated. However, emotional loneliness showed a greater number of significant cross-lagged network connections compared to social loneliness (10 vs 5). Specifically, there were bidirectional and positive associations of emotional loneliness with social loneliness, generalised anxiety, social anxiety, depressive symptoms and paranoid ideation. In turn, higher levels of social loneliness predicted higher levels of social isolation, emotional loneliness and social anxiety. Its higher levels were also predicted by higher levels of emotional loneliness and social isolation.

### Node centralities

**Figure 3.**
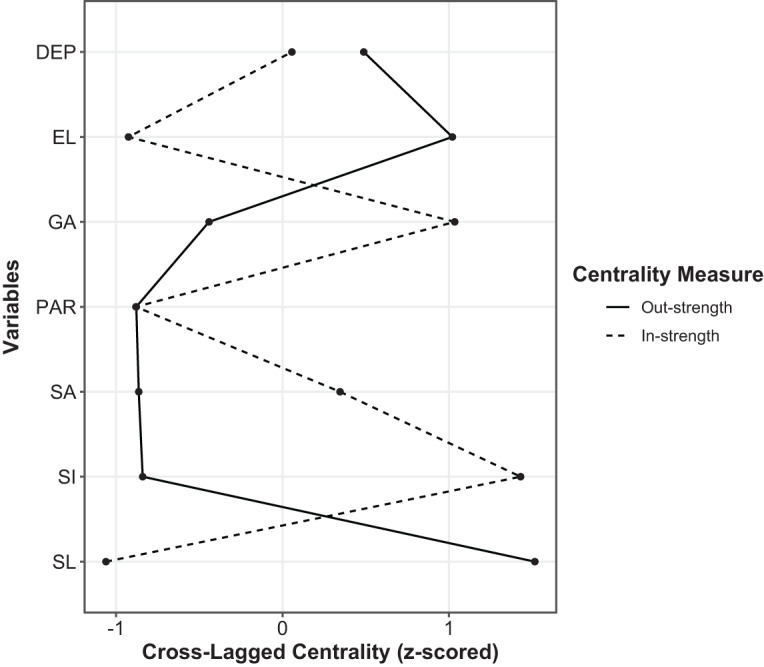
Strength centrality metrics of nodes included in the network. *Note*: DEP, depressive symptoms; EL, emotional loneliness; GA, generalised anxiety; SA, social anxiety; SI, social isolation; SL, social loneliness; PAR, paranoid ideation.

Node centralities are shown in Fig. 3. Social and emotional loneliness had the highest out-strength centrality. In turn, the highest in-strength centrality was found for social isolation and generalised anxiety. However, strength centrality metrics of these nodes were not significantly higher compared to strength centrality metrics of all other nodes in the network (**Supplementary Figs. 3 and 4**).

### Network stability and accuracy

The CS-C values were estimated at 0.750 for edges, 0.622 for out-strength, and 0.479 for in-strength centrality, indicating sufficient stability of a network analysis. These results are visualised in **Supplementary Figs. 5 and 6**.

## Discussion

Main findings from the present study indicate that emotional loneliness might be more robustly related to psychopathological symptoms compared to social loneliness. Specifically, emotional loneliness was bidirectionally associated with all mental health outcomes assessed in the present study. In turn, social loneliness predicted social anxiety but not other mental health outcomes. It was not predicted by any domain of psychopathological symptoms.

It is important to note that the present study operationalised social isolation, loneliness and clinical outcomes as continuous variables. This decision reflects a dimensional perspective on mental health and social functioning, which assumes that differences in symptomatology and connectedness are matters of degree rather than discrete categories (Eaton *et al.*, 2023). Treating these constructs continuously offers greater statistical power, avoids loss of information inherent in dichotomisation, and allows for the detection of associations that extend into the subclinical range. Such variation is meaningful: previous work has demonstrated that even modest reductions in social network size or frequency of contact are associated with increased risk of poorer health outcomes (Steptoe *et al.*, 2013; Valtorta *et al.*, 2016). In the present sample, variation in social isolation and loneliness scores therefore captured both individuals meeting previously proposed cut-offs for and those below these thresholds, enabling us to examine gradients of risk across the full continuum. By modelling outcomes as continuous, we were also able to align our analytic approach with contemporary dimensional models of psychopathology, which emphasise that subclinical symptoms can be prognostically significant for the development of later disorders (Eaton *et al.*, 2023; Hopwood *et al.*, 2023).

The findings align with and expand upon those provided by previous studies that have examined the nuanced roles of different types of loneliness. A recent network analysis of data obtained from university students revealed that emotional loneliness is more closely related to mental outcomes than social loneliness (van den Bulck *et al.*, 2025). Similarly, Wolters *et al.* (2023) revealed some differences between both dimensions of loneliness by showing that emotional loneliness is most strongly correlated with depressive and social anxiety symptoms, while social loneliness is mostly related to social isolation among university students. However, it is needed to note that in this study, both domains of loneliness were associated with depressive and social anxiety symptoms. Generalised anxiety was linked to loneliness through the effects of depressive symptoms. However, some methodological differences between the present study and both network analysis studies should be noted (van den Bulck *et al.*, 2025; Wolters *et al.*, 2023). As opposed to both studies, our analysis focused on cross-lagged associations and covered the levels of paranoid ideation. Moreover, findings from previous network analyses might be relevant for younger populations but not for other age groups. Another study demonstrated that emotional, but not social, loneliness is associated with late-life depression and suicidal ideation (Ngan and Cheng, 2024). Altogether, the findings indicate the importance of perceived intimacy and support the cognitive discrepancy model of loneliness, which underscores the role of social relationship quality in shaping mental health (Perlman and Peplau, 1981).

It is further important to note that social isolation was bidirectionally associated with social, but not emotional, loneliness. This observation supports the theory behind differentiating both dimensions of loneliness, indicating that the overall social network density more closely corresponds with social loneliness. This is also in agreement with the findings from a network analysis by Wolters *et al.,* (2023), who found that social loneliness is mostly explained by social isolation. Moreover, previous studies have shown that loneliness and social isolation are modestly correlated, indicating conceptual differences between both constructs (Cacioppo and Hawkley, 2009; Holt-Lunstad *et al.*, 2015). In this regard, findings from the present study also support the difference between social isolation and loneliness.

Findings from the present study indicate that the recognition of emotional loneliness might be of importance for risk stratification and therapy personalisation. Emotional loneliness may serve as an early indicator of vulnerability to depression, anxiety or paranoid ideation, even in individuals who do not meet diagnostic thresholds. Incorporating brief, validated measures of loneliness into routine primary care, mental health intake assessments and follow-up visits could help clinicians identify at-risk patients who might otherwise remain undetected.

To date, various interventions targeting loneliness have been developed, including those focused on enhancing social networks, general social skills and social cognition (Cacioppo *et al.*, 2015; Hickin *et al.*, 2021; Masi *et al.*, 2011). However, to better understand the relevance of findings from this study for therapy personalisation, it is important to consider the theory behind differentiating social and emotional loneliness. This theory posits that emotional loneliness is not merely a consequence of being alone but of feeling disconnected from those to whom one appears to be emotionally attached (Weiss, 1973). The attachment system, developed in early life, plays a crucial role in determining who is most at risk (Zhang *et al.*, 2022). Therefore, strengthening secure attachments and fostering intimacy in close relationships might be crucial for reducing emotional loneliness and improving mental health. For instance, emotionally focused therapy (EFT) offers a distinctive, attachment-based approach aimed at restoring emotional connection and intimacy – key deficits in emotional loneliness (Mendoza and Leeth, 2025). A recent meta-analysis of nine randomised, controlled trials revealed large effect size estimates of EFT in improving couple distress (Beasley and Ager, (2019). Some studies have also demonstrated the efficacy of EFT among individuals with post-traumatic stress disorder (Weissman *et al.*, 2018) and major depression (Alder *et al.*, 2018; Wittenborn *et al.*, 2019). It has also been suggested that EFT may be effective in alleviating loneliness by strengthening secure attachment bonds and enhancing perceived emotional support (Greenman and Johnson, 2022). However, there is still a scarcity of studies investigating its effects on the level of loneliness. The study by Shahir *et al.,* (2021) investigated the effects of EFT and cognitive behavioural therapy among survivors of intimate partner violence – a population at high risk of loneliness. Both interventions were associated with significant reductions in loneliness. Although EFT did not outperform cognitive behavioural therapy, qualitative data analysis suggested that EFT was uniquely effective in fostering emotional connection and trust, which are the key components of emotional loneliness. Another study, carried out in couples with infertility, revealed beneficial effects of EFT in reducing the level of loneliness (Zamani Zarchi *et al.*, 2020). In turn, individuals experiencing social loneliness may respond better to interventions that expand social networks or enhance social skills.

Beyond clinical implications, these findings may also inform policy and public health practice. Differentiating between social and emotional loneliness suggests that initiatives should not only promote opportunities for social contact, but also create environments that facilitate secure, emotionally supportive relationships. At the community level, this could involve strengthening social prescribing programmes, supporting local initiatives that foster trust and reciprocity, and investing in community spaces that enable meaningful social engagement rather than only casual contact. At the family and couple level, policies that provide resources for relationship counselling, parental support or workplace programmes to improve work–life balance may help individuals maintain emotionally supportive ties. Importantly, the findings suggest that emotional loneliness, often less visible than social isolation, may carry disproportionate risk for poor mental health outcomes. Public health strategies that systematically assess both social and emotional dimensions of loneliness, for example, in routine population health surveys or primary care screening, could therefore improve early identification of individuals at risk. Future research should test whether policy-level interventions that strengthen emotionally supportive environments are effective in reducing loneliness and preventing downstream psychopathology.

There are some limitations of the present study. As with most online population surveys, selection bias cannot be fully excluded. Potential sources include self-selection of individuals with an interest in mental health, underrepresentation of older or less digitally connected groups and attrition between waves. Although participants who completed both assessments did not differ significantly from those lost to follow-up in sociodemographic or clinical characteristics, the sample may nonetheless over-represent individuals with greater digital access, higher health literacy and motivation to participate in research. At this point, it is important to note that problematic internet use has been associated with loneliness (Zhang *et al.*, 2024); however, this phenomenon was not assessed in the present cohort. It should also be noted that mental health outcomes were assessed using self-reports and lacked clinical validation. Moreover, although the questionnaire used to assess loneliness (DJGLS) has been validated, there are concerns related to the fact that negatively and positively worded items are used to assess emotional and social loneliness, respectively (Penning *et al.*, 2014). Also, the response and retention rates (46.4% and 64.2%, respectively) were relatively low. However, a recent meta-analysis revealed the mean response rate in online surveys of 44.1% (Wu *et al.*, 2022). It should be noted that response rates are not always directly related to the validity of findings (Morton *et al.*, 2012), and there is evidence that studies with a response rate of 20% are still able to provide accurate results (Visser *et al.*, 1996). Another limitation is that the study was based on only two measurement time points spanning only 6–7 months. Therefore, the study does not provide insights into long-term trajectories and potential consequences of loneliness. Finally, some limitations of the CLPN approach further need to be recognised (Freichel *et al.*, 2023; Wysocki *et al.*, 2023). Importantly, CLPN does not establish causality, and it assumes that the associations between variables remain consistent across the study period, which may not be the case if these relationships shift over time. The use of regularisation techniques, while preventing overfitting, can bias parameter estimates, and missing data may affect results if attrition is not random. As the current implementation of CLPN is typically restricted to two waves, temporal dynamics may be difficult to disentangle from measurement error.

In conclusion, the findings indicate the rationale behind differentiating emotional and social loneliness. Emotional loneliness might be more closely related to mental health than social loneliness. These findings may be important for risk stratification and personalisation of therapeutic approaches among individuals experiencing loneliness. Replication is needed in studies with longer observation periods and thorough clinical assessment. Future research should also examine the mechanisms linking emotional loneliness to psychopathology, test whether reducing emotional loneliness can alleviate mental health symptoms, and evaluate these processes across different age groups and cultural contexts. Longitudinal and intervention studies will be particularly important to establish directionality and causal pathways.

## Supporting information

10.1017/S2045796025100383.sm001 Misiak et al. supplementary material Misiak et al. supplementary material

## Data Availability

The data supporting the present study are available from the corresponding author upon a reasonable request.

## Supplementary material

The supplementary material for this article can be found at https://doi.org/10.1017/S2045796025100383.
