# Supplementary material for: Unravelling longitudinal associations of social and emotional loneliness with social isolation and mental health outcomes: a cross-lagged panel network analysis
Source: Epidemiol Psychiatr Sci. 2026 Jan 2;35:e1. doi: 10.1017/S2045796025100383 (PMC12816935; doi:10.1017/S2045796025100383)
Supplement: Misiak et al. supplementary material [file S2045796025100383sup001.docx]

**Supplementary Appendix**

**Table 1.** Significant cross-lagged effects observed in the network.

| nodeOut | nodeIn | value |
| --- | --- | --- |
| SL: social loneliness | SI: social isolation | 0.335 |
| DEP: depressive symptoms | GA: generalized anxiety | 0.171 |
| GA: generalized anxiety | DEP: depressive symptoms | 0.130 |
| SL: social loneliness | EL: emotional loneliness | 0.123 |
| EL: emotional loneliness | SL: social loneliness | 0.105 |
| EL: emotional loneliness | GA: generalized anxiety | 0.102 |
| EL: emotional loneliness | SA: social anxiety | 0.099 |
| DEP: depressive symptoms | PAR: paranoid ideation | 0.084 |
| SL: social loneliness | SA: social anxiety | 0.079 |
| EL: emotional loneliness | DEP: depressive symptoms | 0.069 |
| EL: emotional loneliness | PAR: paranoid ideation | 0.061 |
| DEP: depressive symptoms | SA: social anxiety | 0.052 |
| SI: social isolation | SL: social loneliness | 0.036 |
| DEP: depressive symptoms | EL: emotional loneliness | 0.018 |
| PAR: paranoid ideation | GA: generalized anxiety | 0.017 |
| SA: social anxiety | GA: generalized anxiety | 0.016 |
| PAR: paranoid ideation | DEP: depressive symptoms | 0.015 |
| SI: social isolation | SA: social anxiety | 0.015 |
| SA: social anxiety | DEP: depressive symptoms | 0.015 |
| SA: social anxiety | PAR: paranoid ideation | 0.010 |
| PAR: paranoid ideation | SA: social anxiety | 0.007 |
| GA: generalized anxiety | EL: emotional loneliness | 0.004 |
| SA: social anxiety | EL: emotional loneliness | 0.003 |
| PAR: paranoid ideation | EL: emotional loneliness | 0.003 |
| SA: social anxiety | SI: social isolation | 0.002 |

**Figure 1.** The flow diagram of participants.

**
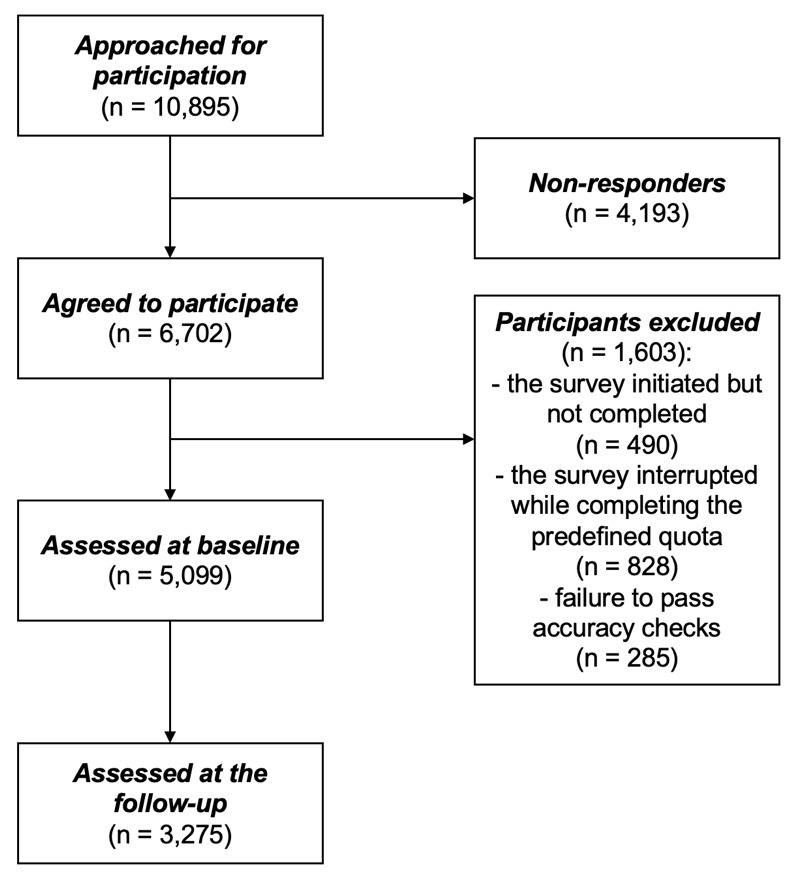
**

**Figure 2.** Bootstrapped differences between cross-lagged edges. Black boxes indicate significant differences between specific edges.

**Figure 3.** Bootstrapped differences between out-strength centrality metrics. Black boxes refer to significant between-node differences.

**Figure 4.** Bootstrapped differences between in-strength centrality metrics. Black boxes refer to significant between-node differences.

**Figure 5.** Stability of strength centrality metrics while dropping various proportion of data from the original dataset. Average correlation of strength centrality metrics with those from the original data set is plotted against the percentage of retained cases.

**Figure 6.** Bootstrapped 95%CI around edge weights.
